# Annual and Seasonal Patterns of Dietary Intake in Australian Adults: A Prospective Cohort Study

**DOI:** 10.3390/nu16162718

**Published:** 2024-08-15

**Authors:** Rachel G. Curtis, Gilly A. Hendrie, Ty Ferguson, Timothy Olds, François Fraysse, Dorothea Dumuid, Wendy J. Brown, Adrian Esterman, Carol A. Maher

**Affiliations:** 1 UniSA Allied Health and Human Performance, Alliance for Research in Exercise, Nutrition and Activity, University of South Australia, Adelaide, SA 5001, Australia; rachel.curtis@unisa.edu.au (R.G.C.); ty.ferguson@unisa.edu.au (T.F.); timothy.olds@unisa.edu.au (T.O.); francois.fraysse@unisa.edu.au (F.F.); dot.dumuid@unisa.edu.au (D.D.); adrian.esterman@unisa.edu.au (A.E.); 2 Health and Biosecurity, Commonwealth Scientific and Industrial Research Organisation (CSIRO), Adelaide, SA 5000, Australia; gilly.hendrie@csiro.au; 3 School of Human Movement and Nutrition Sciences, University of Queensland, Brisbane, QLD 4072, Australia; wbrown@uq.edu.au; 4 Faculty of Health Sciences and Medicine, Bond University, Gold Coast, QLD 4226, Australia

**Keywords:** dietary patterns, adults, season

## Abstract

Poor diet is a major risk factor for non-communicable disease. The aims of this study were to describe temporal patterns and seasonal changes in diet across the year in Australian adults. A total of 375 adults from a prospective cohort study conducted between 1 December 2019 and 31 December 2021 in Adelaide, Australia, were asked to complete the Dietary Questionnaire for Epidemiological Studies at eight timepoints over a year. Average intakes over the previous month of total energy, macronutrients, healthy food groups, and discretionary foods and beverages were derived. Temporal patterns in diet were analysed descriptively. Multilevel linear regression modelling was used to assess seasonal differences in diet. Of the 375 participants recruited, 358 provided sufficient data for analysis. Intake of total energy, all macronutrients, and most discretionary foods and beverages peaked in December. Total energy intake was higher in summer than in autumn, winter, and spring. Fruit intake was higher in summer than in winter. Consumption of alcoholic beverages was higher in summer than in autumn, winter, and spring. Consumption of non-alcoholic beverages was higher in summer than in autumn and winter. This study identified temporal differences in dietary intake among Australian adults. Seasonal effects appear to be driven largely by increases in consumption of foods and beverages over the December (summer) holiday period. These findings can inform the design and timing of dietary interventions.

## 1. Introduction

Poor diet is a risk factor for many non-communicable diseases such as hypertension, cardiovascular disease, stroke, and type 2 diabetes [1,2,3]. In 2017, 11 million deaths globally (approximately one in five deaths) were attributable to dietary risk factors [2]. Although many countries have food-based dietary guidelines [4] and nutrition-related government policies [5], diet quality globally has improved only slightly between 1990 and 2018 and generally remains modest [6]. In Australia, the average diet does not align with the recommendations in the Australian Dietary Guidelines, as people consume too much discretionary food and not enough food from the five healthy ‘core’ food groups (i.e., fruits, vegetables, grains, dairy and alternatives, and meat and alternatives) [7,8]. Effective dietary interventions in Australia, and globally, are needed to increase diet quality and improve health and wellbeing.

Food choices are complex and dynamic, influenced by a range of individual differences (e.g., food preferences) and external factors (e.g., social, cultural, and economic environments) [9]. Included among these are temporal factors, such as cultural events and weather, which may cause variations in diet at different times of the year. Understanding how diet changes over the year is important for delivering interventions at the right time with relevant messages to increase the likelihood of behaviour change.

Previous research has demonstrated that weight gain coincides with key cultural celebrations in the USA, Germany, Japan [10], and Australia [11]. However, longitudinal research examining the impact of cultural and religious events on dietary intake is limited. For example, one study found increases in frequency of unhealthy eating among US adults during the Christmas holiday period [12]. A second study found reduced overall diet quality but no difference in energy intake between the winter holiday period (including Thanksgiving, Christmas, and New Year) and the rest of the year among US midlife women [13]. Research has shown increases in total energy intake during Ramadan among young adults in Algeria [14] and adults in Egypt [15], no change in total energy intake during Ramadan in adults in Lebanon [16], and a decrease in energy intake during Ramadan among Malaysian adults [17].

There is also inconsistent evidence regarding seasonal differences in dietary intake. Some studies report higher energy intake in winter than in summer among Spanish men [18], Japanese women [19], and middle-aged and elderly women from the USA [20]. Yet other research shows no seasonal differences in energy intake among men from Israel [21], women from the USA [13], or adults from the Netherlands [22], Brazil [23], or the USA [24]. Additionally, some studies have shown seasonal differences in fat intake [19,21,22,25], whilst others have not [23,24]. Research examining food intake generally shows seasonal variation in at least some foods [13,18,21].

The variability in dietary patterns underscores the need for region-specific data. In Southern Hemisphere countries like Australia, the Christmas holiday period coincides with summer, potentially affecting dietary patterns differently from the Northern Hemisphere, where these celebrations occur in winter. Cross-sectional data in Australia indicate higher intake of total energy and most food groups in December and January compared to November, but there was no direct comparison between summer and winter months [26]. A 1990 longitudinal study did not find seasonal variations in energy intake among Australian adults but did note differences in macronutrient intake among men (saturated fat intake was higher in autumn and spring than in summer and sugar was higher in spring than in winter) [27]. There were insufficient timepoints to examine other annual patterns (e.g., the Christmas holiday period) and dietary patterns may have changed since 1990, when the study was conducted. The aim of the present study was to describe variation in dietary intake over a 13-month period in Australian adults between 2019 and 2021. Specifically, the aims were as follows:

1. To describe the patterns in diet (total energy intake, macronutrient intake, healthy food groups, and discretionary foods and beverages) across the year;
2. To determine whether dietary intake varies by season.

## 2. Materials and Methods

### 2.1. Study Design

This study used data from the Annual Rhythms in Adults’ lifestyle and health (ARIA) cohort study. ARIA collected data on body weight, 24 h movement behaviours, dietary intake, and wellbeing in a cohort of Australian adults over a 13-month period to assess temporal trends across a year. Full details of the study protocol have been published elsewhere [28]. Ethics approval was provided by the University of South Australia Human Research Ethics committee (protocol number: 201901). The study was prospectively registered on the Australian New Zealand Clinical Trial Registry (ACTRN12619001430123).

### 2.2. Setting and Participants

A community-based sample of 375 adults was recruited from the greater metropolitan area of Adelaide, South Australia. Participants were parents or guardians of children enrolled in a separate cohort study, “Life on Holidays” [29] (cohorts 1 and 2) or parents of primary school-aged children recruited through general advertising (e.g., mainstream and social media; cohort 3). Participants were recruited in 2 waves: cohort 1 commenced on 1 December 2019 and were followed up until 31 December 2020, and cohorts 2 and 3 commenced on 1 December 2020 and were followed up until 31 December 2021.

Inclusion criteria were being 18 to 65 years old, being a parent or guardian of a primary school-aged child enrolled in the Life on Holidays study [29] or of a child aged 5 to 12 years, residing in greater metropolitan Adelaide, having access to a Bluetooth-enabled mobile device or computer with home internet, understanding English, and being ambulant. Exclusion criteria were pregnancy, having an implanted electronic medical device, or experiencing or receiving treatment for any life-threatening condition which impacted daily lifestyle and health.

A baseline home visit was conducted prior to study commencement, where participants had their height measured and were given a Fitbit Aria body weight scale (Aria 2 or Aria Air scale; Fitbit Inc., San Francisco, CA, USA) and a Fitbit Charge 3 activity monitor (no activity data were used in the present analysis) and instructed on their use. Participants completed a baseline demographic survey (e.g., age, sex, education, marital status, Aboriginal and Torres Strait Islander status, country of birth, smoking status and chronic conditions) either prior to or during the home visit. They also completed eight online surveys to assess the previous month’s dietary intake in early December (baseline), January, March, April, June, August, October, and the following December. Therefore, we obtained dietary data for the months of November (first November), December, February, March, May, July, September, and November (second November). We will henceforth refer to the months for which dietary data are available, rather than the months the surveys were completed. These timepoints were chosen to assess seasons and key periods of the year (e.g., Christmas), as outlined in the study protocol [28].

At study completion, participants kept the Fitbit equipment and received an AUD 100 honorarium.

### 2.3. Variables

#### 2.3.1. Dietary Intake

Dietary intake was assessed at each timepoint using the Dietary Questionnaire for Epidemiological Studies (Version 3.2) developed by the Cancer Epidemiology Division, Cancer Council Victoria [30,31], which was modified to ask participants about their dietary intake over the previous month instead of the previous 12 months. At each timepoint, the Dietary Questionnaire for Epidemiological Studies (DQES) provided an estimate of average daily consumption over the previous month for total energy (kJ), macronutrients (g), and individual foods (g) based on 144 food and beverage items, using nutritional information from the NUTTAB 2010 [32] and AUSNUT 2007 databases [33]. The DQES has good reproducibility and good agreement with weighed food records [34,35,36].

Grams per day of macronutrients (protein, fat, carbohydrate including fibre, and alcohol) were converted to kJ/day using standard conversion factors of 17 kJ per gram for protein, 37 kJ per gram for fat, 17 kJ per gram for carbohydrate, 8 kJ per gram for fibre, and 29 kJ per gram for alcohol [37,38]. Individual foods were grouped into six healthy food groups—fats and oils, dairy, grains, meat and meat alternatives (i.e., seafood, nuts and eggs), fruits, and vegetables—and five discretionary groups: sweet discretionary foods (e.g., confectionery and sweet pastries), savoury discretionary foods (e.g., processed meats and savoury pastries), alcoholic beverages, non-alcoholic beverages (i.e., soft drinks and juices; not including water), and miscellaneous (e.g., salad dressings, spreads, tea and coffee). Total intake for each food group was estimated in grams per day. 

#### 2.3.2. Participant Descriptive Characteristics

Height was measured at the baseline home visit using a portable stadiometer (Leicester Height Measure MKII; Invicta Plastics, Leicester, England) according to International Society for the Advancement of Kinanthropometry assessment procedures [39]. Baseline weight (participants’ first valid weight measurement from the Fitbit Aria body weight scale) was collected remotely using bespoke Fitnesslink software (Version 1, Portal Australia, Adelaide, Australia). Weight status was classified using body mass index (kg/m^2^) as underweight (<18.5), normal weight (18.5 to <25), overweight (25 to <30) or obese (≥30). Self-reported participant characteristics included date of birth, sex, smoking status, Aboriginal and Torres Strait Islander status, country of birth, marital status, education, and chronic conditions. Percentage of energy from protein, fat, carbohydrate, and alcohol was calculated at baseline.

### 2.4. Statistical Analysis

Demographic and baseline data were analysed descriptively using means and standard deviations for continuous measures, and counts and percentages for categorical measures. For each participant, the overall yearly means for total energy intake, macronutrients, healthy food groups and discretionary foods and beverages were calculated as the mean over all timepoints. The difference between each timepoint and this yearly mean was then calculated for each participant. Differences in food group consumption were calculated as a percentage of each participant’s yearly mean. The yearly mean and the difference from the yearly mean at each timepoint were then averaged over all participants, graphed, and visually inspected. Yearly patterns including maximum variation (i.e., peak-to-trough differences) were described. 

Multilevel linear regression modelling was used to compare energy intake, macronutrients, healthy food groups, and discretionary foods and beverages across seasons. Multilevel modelling with random intercepts was used to adjust for the non-independence of the data and to account for nesting of repeated measures within individuals, individuals within families, and families within waves. July data were used for the winter season, and for the other three seasons, the average of the first and last month of the season were used (summer: mean of December and February; autumn: mean of March and May; winter: July; spring: mean of September and November). Dietary variables were included as dependant variables in separate models, with season included as a fixed effect. No covariates were included. Analyses were completed in Stata version 17 (StataCorp, College Station, TX, USA). Statistical significance was set at α = 0.05 with sequential Holm–Bonferroni adjustments [40] applied to pairwise comparisons.

## 3. Results

### 3.1. Participants

Ninety-five percent of participants (358/375) provided demographic data and completed at least one dietary survey. Of the 358 participants whose data were analysed, 89% (319) completed at least six dietary surveys (median = eight out of eight possible surveys, IQR = 7–8). Table 1 shows participant characteristics at baseline. Just over half of participants (57%) were female, with an average age of 40 years (range of 27 to 65 years). Most were born in Australia. Approximately one-third of participants were in each of the normal, overweight, and obese weight status categories. Almost half the participants had a university degree. More than half reported one or more chronic illness and fewer than 1 in 10 smoked. On average, participants’ energy intake was 7683 kJ/day (1836 kcal/day), comprising 19% protein, 37% fat, 40% carbohydrate, and 3% alcohol at baseline.

### 3.2. Dietary Patterns across the Year

**Figure 1:**
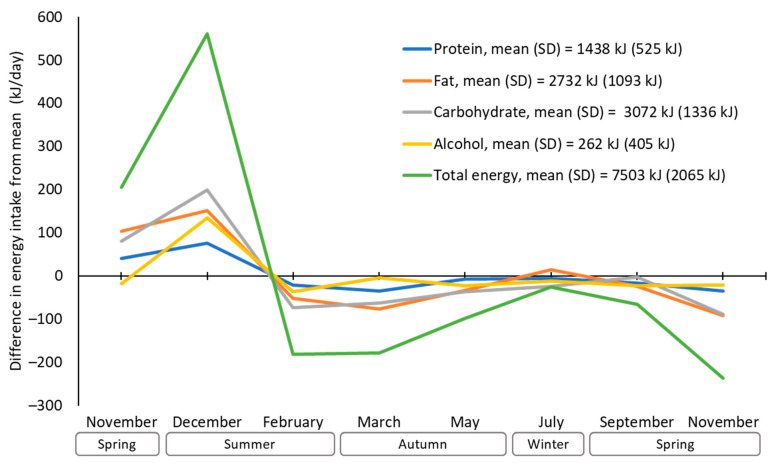
Difference in total energy and macronutrient intake (kJ/day) from yearly mean.

Average differences in total energy and macronutrient intake from each participant’s yearly means are shown in Figure 1. Peak-to-trough variation (i.e., December compared with the second November) in total energy intake was approximately 800 kJ (191 kcal, 11% of mean energy intake). A spike in December (560 kJ more than average) was followed by a decline in February (180 kJ less than average). There was less variation between February and the following second November (approx. 210 kJ), with total energy intake remaining lower than average despite a slight increase between March and July. On average, peak-to-trough variations for protein, fat, carbohydrate, and alcohol were approximately 112 kJ (8%), 242 kJ (9%), 288 kJ (10%) and 170 kJ (65%), respectively, and generally followed similar patterns to total energy intake. Carbohydrate and alcohol intakes showed the largest increases between November and December.

**Figure 2:**
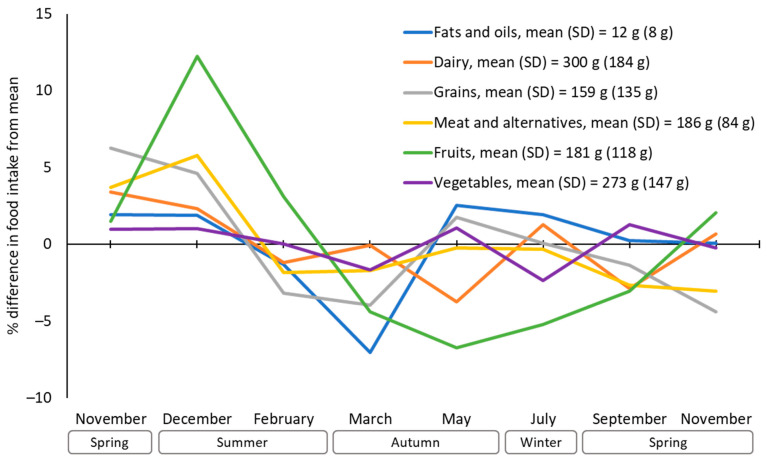
Percentage difference in intake of healthy food groups from yearly mean.

Figure 2 shows the average percentage differences in intake of healthy food groups from each participant’s yearly means. The average peak-to-trough variations were 19% for fruits, 11% for grains, 10% for fats and oils, 9% for meat and meat alternatives, 6% for dairy and 4% for vegetables. Consumption of healthy food groups was generally above average in the first November and December, and below average in February and March. Notably, fruit was highest in December and lowest in May and July.

**Figure 3:**
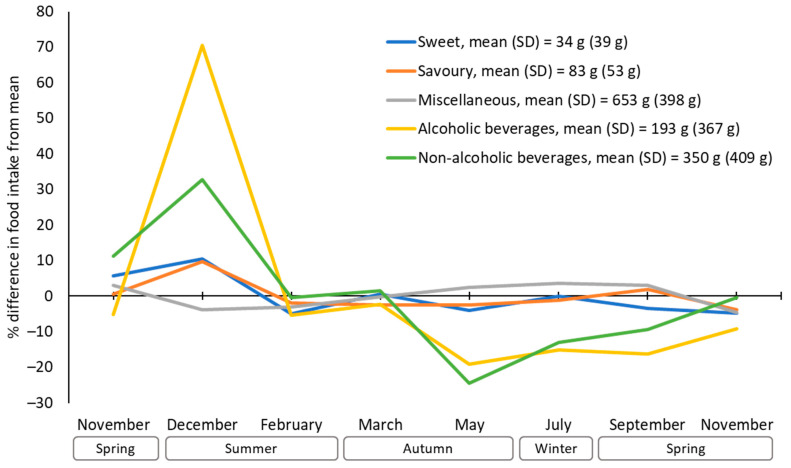
Percentage difference in discretionary food intake from yearly mean.

Figure 3 shows the average percentage differences in intakes of discretionary foods and beverages from each participants’ yearly means. The peak-to-trough variations were 90% for alcoholic beverages, 57% for non-alcoholic beverages, 15% for sweet discretionary foods, 13% for savoury discretionary foods, and 8% for miscellaneous discretionary foods. Intakes of discretionary foods were generally above average in the first November and December and below average for the rest of the year, except for miscellaneous discretionary foods. Alcoholic and non-alcoholic beverages showed a spike in December followed by a decline in February. There was a second decline in May, followed by a gradual increase for the remainder of the year.

### 3.3. Seasonal Differences in Dietary Patterns

Results from multilevel modelling analyses of seasonal differences in total energy and macronutrient intake are shown in Table 2. There were no seasonal differences in carbohydrate intake. Total energy, protein, and alcohol intakes were higher in summer than in autumn (total energy +345 kJ, 5%; protein +61 kJ, 4%; alcohol +63 kJ, 20%), winter (total energy +250 kJ, 3%; protein +50 kJ, 3%; alcohol +66 kJ, 21%), and spring (total energy +357 kJ, 5%; protein +67 kJ, 5%; alcohol +77 kJ, 25%). Fat intake was higher in summer than autumn (+111 kJ, 4%) and spring (+116 kJ, 4%). There were no other significant pairwise comparisons.

Results from multilevel modelling analyses of seasonal differences in consumption of healthy food groups are shown in Table 3. There were no seasonal differences in fats and oils, dairy, grains, or vegetables. Intake of meat was high in summer than in winter (+10 g, 5%) and spring (+13 g, 7%). Intake of fruit was higher in summer than in winter (+18 g, 10%). There were no other significant pairwise comparisons.

Results from multilevel modelling analyses of seasonal differences in consumption of discretionary foods and beverages are shown in Table 4. There were no seasonal differences in sweet discretionary foods. Savoury discretionary foods were higher in summer than in autumn (+7 g, 8%). Alcoholic beverages were higher in summer than in autumn (+40 g, 18%), winter (+51 g, 23%), and spring (+57 g, 26%). Non-alcoholic beverages were higher in summer than in autumn (+80 g, 22%) and winter (+75 g, 20%). Miscellaneous discretionary foods were lower in summer (−48 g, −8%) than in winter. There were no other significant pairwise comparisons.

## 4. Discussion

The aim of this study was to describe patterns in diet (total energy intake, macronutrient intake, healthy food groups and discretionary foods and beverages) across the year, and examine seasonal differences in dietary intake. Dietary intake showed variability across the year, with consumption of total energy, macronutrients, and many food groups being highest in December. Seasonal analyses showed that intake of total energy, fat, protein, and alcohol was highest in summer. Intake of meat, fruit, alcoholic and non-alcoholic beverages was higher in summer than winter. There were no significant seasonal variations in intake of fats and oils, dairy, grain, vegetables, and sweet discretionary foods.

Before considering the implications of these findings, the strengths and limitations of the study must be acknowledged. A major strength of this study is the prospective longitudinal research design with repeated measures assessing dietary intake across all four seasons, as well as the December holiday period. To our knowledge, this is the first Australian study to do this. We note, however, the limitations of self-report dietary recall using a food frequency questionnaire, including the potential omission of some foods and participant underreporting. On average, participants reported consuming 7683 kJ/day (1836 kcal/day) at baseline, which would be an underestimate for most middle-aged adults [41]. Energy intake in the second November was lower than the first November, suggesting that underreporting might have been more prevalent in the later surveys. Although the DQES has been shown to have good agreement with weighed food records, its ability to detect changes in diet, and therefore differences in intake between seasons, is unknown. Additionally, although our sample was broadly representative of the Australian adult population in terms of sex (57% female vs. 51% of 35- to 44-year-old Australian adults [42]), body mass index (68% overweight or obese vs. 66% of 35- to 44-year-old Australian adults [43]), and smoking status (9% vs. 11% of Australian adults [44]), our sample consisted predominantly of residents from a single Australian city and only included parents of school-aged children (vs. 77% of Australian women aged 30 to 49 years who report having children [45]). While our sample had somewhat higher rates of university education than the general Australian adult population, it still included a reasonable mix of education levels. These factors may limit the generalisability of our findings, especially as others have shown that diets may differ between parents and non-parents, especially in relation to fast food intake [46,47], and parents of primary school-aged children may be younger than the overall general adult population. The data collection period overlapped with the COVID-19 pandemic, which could have influenced eating behaviours due to restrictions on gatherings and restaurant operations, although the impact in our study region was less severe than other areas.

Energy intake increased in December, which appears to be driven mostly by an increase in carbohydrate, fat and alcohol consumption. Additionally, energy and macronutrient (except carbohydrate) intakes were highest in summer, which contrasts with the notion that people eat less in hot weather [48]. However, given that the spike in December was followed by a dip in February, seasonal differences appear to be driven more by the holiday period than the weather.

Most discretionary foods (alcoholic drinks, non-alcoholic drinks, savoury and sweet foods) also peaked in December. The increases in energy and discretionary food and beverage consumption seen in December are likely due to the increased frequency of social gatherings during the holiday period, as people tend to eat more while eating with others than when eating alone [49] and report that enjoying a special occasion is the most common reason for unhealthy snacking [50]. Seasonal analyses also showed that alcohol intake was highest in summer. Research in the northern hemisphere (i.e., Sweden and Scotland) has shown higher alcohol consumption in summer, as well as a second peak in December, during the winter Christmas holiday period [51,52]. The single December/summer spike seen in our study may be explained by the co-occurrence of summer and the Christmas period in Australia. Despite a small December peak coinciding with the holiday season, there were few seasonal differences in savoury discretionary foods (higher in summer than autumn) and no seasonal differences in sweet discretionary foods. Among Australian adults, one-third of energy comes from discretionary foods [8] and availability of these foods does not differ by season. A lack of seasonal effects may therefore reflect a consistent intake of foods such as processed meats, sweet and savoury pastries, and confectionary. The miscellaneous category showed opposite patterns to the other discretionary foods; it showed no December spike and was higher in winter than in summer. This may be because it includes hot drinks such as tea and coffee that people tend to consume more in winter [53].

Patterns in consumption of healthy foods were less consistent than discretionary foods. Fruit consumption was slightly higher in summer than in winter, with a peak in December. The December peak might reflect consumption of fruit-based snacks and desserts during holiday celebrations. The dip in winter might be explained by the cost of preferred fruits; though globalisation means that most foods are available all year across Australia, certain fruits such as stone fruits and grapes are not grown in Australia in winter [54] and may therefore be more expensive.

### Implications

The results from this study highlight the December holiday period as a key period for dietary intervention. Popular rhetoric suggests that overindulging during the Christmas period does not have significant health effects. However, even a short period of increased energy intake could lead to weight gain, which could accumulate each year if there is not sufficient compensation at other times. Indeed, previous research in the USA showed that winter holiday weight gain was not reversed during the subsequent spring and summer months [55]. In our study, consuming approximately 570 additional kJ per day (136 kcal/day) in December equates to approximately 0.5 kg of weight gain (assuming 32.2 MJ per kg weight gain, without accounting for physiological adaptations [56]), which is similar to the 0.5 kg annual weight gain previously estimated in Australian adults [57,58]. The increase in energy intake in December appears to be driven by a range of foods; therefore, intervention messaging could focus generally on reducing overeating during holiday celebrations or on eating foods that are lower in kJ, such as vegetables, to reduce the impact of overeating.

On average, participants drank three more standard drinks of alcohol per week in December than their yearly average. Alcohol consumption is known to contribute to many health conditions [59,60]. Though the average participant in our study met the Australian government guidelines of no more than 10 standard drinks per week [60] throughout the year, including in December, the holiday period may lead to drinking that exceeds the guidelines for some individuals. Interventions promoting healthy drinking behaviour during this time may be warranted. Given that there was also an increase in consumption of non-alcoholic beverages (i.e., soft drinks and juices) in December, intervention messaging should ideally promote low-kilojoule beverages and water, rather than higher-kilojoule beverages like sugar-sweetened drinks.

Finally, our findings highlight the importance of accounting for temporal variations when designing research protocols. Assessments conducted at different times of the year could significantly influence dietary findings, suggesting that seasonality should be considered in dietary research and interventions.

## 5. Conclusions

This study examined dietary patterns in Australian adults and identified temporal differences in dietary intake. The results highlighted increased intake of energy, macronutrients, alcohol, and some foods in December. These findings suggest that the December holiday period, a time traditionally associated with increased social gatherings and festive celebrations, plays a pivotal role in influencing dietary behaviours. The variability in diet throughout the year highlights the importance of considering seasonal effects when conducting dietary assessments and planning interventions. There is a strategic opportunity to implement dietary interventions during the holiday season to prevent the typical weight gain associated with increased energy intake. To optimise public health outcomes, future research should explore the efficacy of such interventions and develop guidelines that accommodate seasonal variations in dietary habits.

## Figures and Tables

**Table 1:** Participant characteristics at baseline (*n* = 358 ^a^).

	Mean	(SD)
Age (years)	40.2	(5.9)
Weight at baseline (kg)	83.7	(20.2)
Height (cm)	170.3	(9.5)
Total energy intake (kJ/day)	7683	(3222)
Percentage of energy intake		
Energy from protein (%)	19.4	(3.8)
Energy from fat (%)	36.9	(5.8)
Energy from carbohydrate (%)	40.4	(7.5)
Energy from alcohol (%)	3.2	(5.3)
	*n*	(%)
Sex		
Female	203	(57)
Male	155	(43)
Weight status category		
Underweight	1	(0.3)
Normal	112	(31.2)
Overweight	121	(33.7)
Obese	124	(34.5)
Smoker	32	(8.9)
Aboriginal and Torres Strait Islander peoples	4	(1.1)
Born in Australia	274	(76.5)
Marital status		
Married/de facto	305	(85.2)
Separated, divorced or widowed	28	(7.8)
Never married	25	(7.0)
Education		
Year 10 or less	17	(4.8)
Year 11–12	48	(13.4)
Certificate/Diploma	120	(33.6)
University degree	173	(48.3)
Chronic conditions		
None	156	(43.6)
Single	106	(29.6)
Multiple	96	(26.9)

^a^ *n* = 337 for total energy intake (kJ/day) and percentage of energy intake from protein, fat, carbohydrate, and alcohol.

**Table 2:** Estimated marginal means and pairwise comparisons from multilevel modelling analyses of seasonal differences in total energy and macronutrient intake (kJ/d; *n* = 345).

	Season	Estimated Marginal Means	[95% CI]	Group	
Total energy	Summer	7582.8	[7316.5, 7849.2]	A	
	Autumn	7238.2	[6970.4, 7506.0]		B
	Winter	7332.8	[7066.7, 7598.9]		B
	Spring	7225.4	[6956.6, 7494.1]		B
Protein	Summer	1456.7	[1403.1, 1510.3]	A	
	Autumn	1395.4	[1341.5, 1449.3]		B
	Winter	1406.3	[1352.8, 1459.9]		B
	Spring	1389.5	[1335.3, 1443.6]		B
Fat	Summer	2746.5	[2637.5, 2855.6]	A	
	Autumn	2636.0	[2526.3, 2745.7]		B
	Winter	2700.6	[2591.6, 2809.5]	A	B
	Spring	2630.8	[2520.7, 2740.9]		B
Carbohydrate	Summer	3064.2	[2945.4, 3182.9]	A	
	Autumn	2952.7	[2833.2, 3072.1]	A	
	Winter	2975.7	[2857.1, 3094.2]	A	
	Spring	2967.1	[2849.1, 3087.1]	A	
Alcohol	Summer	308.7	[259.0, 358.4]	A	
	Autumn	245.5	[195.6, 295.5]		B
	Winter	243.2	[193.5, 292.9]		B
	Spring	231.9	[181.7, 282.0]		B

Note: Margins sharing a letter in the group column are not significantly different using Holm–Bonferroni adjusted significance levels. All models include random intercepts to account for the nested data structure.

**Table 3:** Estimated marginal means and pairwise comparisons from multilevel modelling analyses of seasonal differences in intake of healthy food groups (g/d; *n* = 345).

	Season	Estimated Marginal Means	[95% CI]	Group	
Fats and oils	Summer	12.0	[11.1, 12.9]	A	
	Autumn	11.8	[10.9, 12.7]	A	
	Winter	12.1	[11.2, 13.0]	A	
	Spring	11.9	[11.00, 12.8]	A	
Dairy	Summer	299.8	[277.7, 321.8]	A	
	Autumn	298.0	[275.8, 320.1]	A	
	Winter	299.8	[277.8, 321.9]	A	
	Spring	302.7	[280.4, 325.0]	A	
Grains	Summer	154.9	[143.8, 165.9]	A	
	Autumn	152.26	[141.0, 163.3]	A	
	Winter	151.2	[140.2, 162.3]	A	
	Spring	151.5	[140.4, 162.7]	A	
Meats	Summer	189.8	[179.6, 200.1]	A	
	Autumn	180.6	[170.3, 190.8]	A	B
	Winter	179.6	[169.4, 189.8]		B
	Spring	177.0	[166.7, 187.4]		B
Fruits	Summer	187.8	[173.4, 202.1]	A	
	Autumn	172.4	[157.9, 186.9]	A	B
	Winter	169.8	[155.5, 184.1]		B
	Spring	182.5	[167.9, 197.0]	A	B
Vegetables	Summer	273.5	[255.2, 291.7]	A	
	Autumn	273.2	[254.9, 291.6]	A	
	Winter	270.8	[252.6, 289.0]	A	
	Spring	274.1	[255.6, 292.5]	A	

Note: Margins sharing a letter in the group column are not significantly different using Holm–Bonferroni adjusted significance levels. All models include random intercepts to account for the nested data structure.

**Table 4:** Estimated marginal means and pairwise comparisons from multilevel modelling analyses of seasonal differences in discretionary food intake (g/d; *n* = 345).

	Season	Estimated Marginal Means	[95% CI]	Group	
Sweet discretionary	Summer	31.6	[27.2, 36.1]	A	
	Autumn	31.5	[27.1, 36.0]	A	
	Winter	32.6	[28.2, 37.0]	A	
	Spring	31.9	[27.4, 36.3]	A	
Savoury discretionary	Summer	84.7	[79.1, 90.2]	A	
	Autumn	77.7	[72.1, 83.3]		B
	Winter	82.1	[76.5, 87.6]	A	B
	Spring	79.7	[74.1, 85.3]	A	B
Alcoholic beverages	Summer	218.0	[179.5, 256.5]	A	
	Autumn	177.8	[139.1, 216.5]		B
	Winter	167.0	[128.5, 205.5]		B
	Spring	161.1	[122.2, 200.0]		B
Non-alcoholic beverages	Summer	364.6	[322.0, 407.2]	A	
	Autumn	285.0	[242.0, 328.1]		B
	Winter	289.8	[247.3, 332.4]		B
	Spring	322.3	[278.9, 365.6]	A	B
Miscellaneous	Summer	625.5	[581.6, 669.5]	A	
	Autumn	649.3	[605.2, 693.5]	A	B
	Winter	673.7	[629.8, 717.6]		B
	Spring	646.8	[602.5, 691.0]	A	B

Note: Margins sharing a letter in the group column are not significantly different using Holm–Bonferroni adjusted significance levels. All models include random intercepts to account for the nested data structure.

## Data Availability

Data used in the current study may be obtained from the corresponding author upon reasonable request. The data are not publicly available due to restrictions imposed by the ethics restrictions, as participants did not provide consent for public data sharing.
